# National disparities in access to physical therapy after rotator cuff repair between patients with Medicaid vs. private health insurance

**DOI:** 10.1016/j.jseint.2020.11.006

**Published:** 2021-01-16

**Authors:** Emily J. Curry, Ian R. Penvose, Brock Knapp, Robert L. Parisien, Xinning Li

**Affiliations:** a Department of Biostatistics and Epidemiology, Boston University School of Public Health, Boston, MA, USA; b Department of Orthopaedic Surgery, Boston University School of Medicine, Boston, MA, USA; c Department of Orthopaedic Surgery, Harvard Medical School, Boston, MA, USA

**Keywords:** Rotator cuff Repair, Physical therapy, Insurance status, Affordable Care Act, Access to care, Medicaid

## Abstract

**Background:**

Arthroscopic rotator cuff repair is an effective treatment for patients with symptomatic rotator cuff tears. Ensuring timely and appropriate postoperative access to physical therapy (PT) is paramount to the achievement of optimal patient outcomes. Extended immobility due to a lack of formal rehabilitation can lead to decreased range of motion, continued pain, and potential reoperation for stiffness. The purpose of this study is to evaluate national disparities in access to PT services after rotator cuff repair between patients with private vs. Medicaid insurance. This study will further evaluate differences in access to PT services between states that have previously undergone Medicaid expansion as compared with those states which have not.

**Methods:**

The American Physical Therapy Association Website was used to identify 10 physical therapy practices from the capital city in every state. Each physical therapy practice was contacted using a mock-patient script for a patient with Medicaid insurance or private (Blue Cross Blue Shield) insurance. To maintain anonymity, calls were made by two separate investigators. Univariate analysis included independent sample t-test for differences between the study groups for continuous variables. Chi square or Fisher's exact test assessed differences in discrete variables between the study groups.

**Results:**

Contact was made with 465 of 510 (91.2%) physical therapy practices. Overall, 52.7% accepted Medicaid insurance, while 94.9% accepted private insurance (*P* < .001). Medicaid insurance was more likely to be accepted in a Medicaid expansion state than a nonexpansion state (56.1% vs. 46.3%, *P* = .05). Private insurance was also more likely to be accepted in a Medicaid expansion state than a nonexpansion state (96.7% vs. 91.3%, *P* = .01). The time to first appointment varied more in Medicaid expansion states (private range: 0-43 days, Medicaid range: 0-72 days) than in nonexpansion states (private range: 0-11 days, medicaid range: 0-10 days).

**Conclusion:**

Significantly fewer PT practices accepted Medicaid insurance nationally compared with private insurance, which suggests that patients with Medicaid insurance have greater difficulty accessing PT after rotator cuff repair in the United States compared with patients with private insurance. While Medicaid insurance was more likely to be accepted in a Medicaid expansion state, this finding was only borderline significant, which indicates that patients in Medicaid expansion states are still having difficulty accessing PT, despite efforts to expand government insurance coverage to improve access to care. Orthopedic surgeons should counsel their patients with Medicaid insurance to seek out PT as early as possible in the postoperative period to avoid delays in rehabilitation.

Arthroscopic rotator cuff repair (RCR) is an effective treatment for patients with symptomatic full-thickness rotator cuff tears, which are known to be one of the most common sources of shoulder pain and dysfunction.^11^^,^^12^ Ensuring timely and appropriate access to physical therapy (PT) services in the postoperative period after RCR is paramount to the achievement of optimal patient outcomes and avoidance of delayed restoration of range of motion and function.^3^^,^^8^ Extended immobility due to a lack of formal rehabilitation can lead to decreased range of motion, continued pain, and potential reoperation for lysis of adhesions.

The Patient Protection and Affordable Care Act (PPACA) enacted in 2010 provides access to healthcare services through expanded health insurance options for those patients who lack health coverage.^9^ In particular, the PPACA enabled states to opt into expanded Medicaid programs, which provided wider eligibility criteria for low-income patients to receive Medicaid insurance.^10^ However, in 2012, the Supreme Court ruled that states could not be required to opt into expanded Medicaid programs. Therefore, Medicaid expansion through the PPACA is dictated on a state-by-state basis.^1^ States opting into Medicaid expansion realized a 10% reduction in uninsured nonelderly adults as compared with only a 4% reduction in nonexpansion states between 2013 and 2017. While the PPACA has provided increased access to health insurance, large disparities still exist between privately and publicly insured patient populations in the United States.

A recent study evaluating access to PT services after RCR in the Greater Boston Area found that only 51.4% of PT practices accepted Medicaid as compared with 96.4% accepting private insurance.^7^ Among those practices accepting Medicaid insurance, Medicaid patients experienced significantly longer wait times as compared with privately insured patients. This finding is particularly concerning as Massachusetts has one of the most robust Medicaid expansion plans in the United States.

The purpose of this study is to evaluate national disparities in access to PT services after RCR between patients with private vs. Medicaid insurance. This study will further evaluate differences in access to PT services between states that have opted to expand Medicaid services as compared with those states which have not. Our primary study objectives are to 1) determine the number of PT practices nationwide that will accept Medicaid vs. private insurance and 2) determine whether there are differences in access to care for Medicaid vs. privately insured patients based on the state's Medicaid expansion status. We hypothesize private insurance will be accepted more often at PT clinics than Medicaid insurance in both Medicaid expansion and nonexpansion states.

## Methods

The American Physical Therapy Association (APTA) was used to randomly select and identify 10 PT practices from the capital city in every state and Washington DC (N = 510). The list was cross-referenced with Yelp and Google Reviews to ensure that the practice is current and credible. Credibility was determined by the practice having a minimum of 2 reviews on Yelp or Google Reviews. The use of Yelp and Google Reviews was to not only quantify patient satisfaction with a particular practice but also to ensure practices selected from the APTA were currently open and available for new patients. If a capital city did not contain an adequate number of PT practices, the remaining number of practices needed to reach a total of 10 in each state were obtained from the next largest city in that particular state. For example, if Juneau, Alaska did not contain an adequate number of PT practices, the remaining PT practices were identified in Anchorage. Once the PT practices in each state were identified, each PT practice was contacted with utilization of a mock script (Appendix 1) for a patient with Medicaid Insurance or Blue Cross Blue Shield insurance (private insurance). To maintain anonymity, calls were placed by two separate investigators. It was randomized via coin flip to determine which “patient” would be contacting the PT clinic first: the private insurer or the Medicaid insurer. Once the first call was placed by either mock patient, there was a minimum of a 1-week waiting period before the next mock patient could call. Two attempts were made to reach a given PT practice and if there was no answer on the second attempt, the practice was excluded. For practical reasons, no voice messages were left.

If a practice did not accept a particular insurance type (Medicaid or private insurance), the caller inquired as to the reason for not accepting the insurance plan. Reasons were categorized into one of six groups: 1) unknown reason, 2) accept insurance plan only as secondary insurance, 3) only pediatric patients accepted on the insurance plan, 4) the clinic did not have a contract with a given insurance carrier, 5) low or no payment from the insurance plan, or 6) practice only accepted cash payment and did not contract with insurance plans.

States were classified as being a Medicaid expansion state or a non-Medicaid expansion state with regards to their Medicaid expansion status as of April 2018. At the time of the survey, 64.7% of states had undergone Medicaid expansion. This included the states of Alaska, Arizona, Arkansas, California, Colorado, Connecticut, Delaware, Hawaii, Illinois, Indiana, Iowa, Kentucky, Louisiana, Maine, Maryland, Massachusetts, Michigan, Minnesota, Montana, Nevada, New Hampshire, New Jersey, New Mexico, New York, North Dakota, Ohio, Oregon, Pennsylvania, Rhode Island, Vermont, Washington, Washington D.C., and West Virginia. Conversely, the following states (35.3%) were not part of Medicaid expansion: Alabama, Florida, Georgia, Idaho, Kansas, Mississippi, Missouri, Nebraska, North Carolina, Oklahoma, South Carolina, South Dakota, Tennessee, Texas, Utah, Virginia, Wisconsin, and Wyoming. The ZIP code of each PT practice was recorded and the US Census household median income for the ZIP code in which the PT practice was located was recorded. The weighted Yelp score was calculated by the total number of patient reviews and overall rating (0 to 5) for each PT practice at the time of contact. States were then reclassified by the US Census Bureau's definition of region: Northeast, Midwest, South, and West. Additional data collected from each PT clinic included time to first appointment for the patient and whether the PT clinic was able to refer the patient to another clinic if they did not accept the patient's specific form of insurance.

Descriptive analyses of the PT practices were conducted. Descriptive analyses consisted of calculating mean and standard deviations for continuous variables. Frequencies and percentages were calculated for categorical or discrete variables. Univariate analysis included independent-sample t-test for differences between insurance groups (Medicaid vs. BCBS) and Medicaid expansion status (expansion state vs. nonexpansion state) with continuous variables. Chi square test was used to assess differences in discrete variables between the aforementioned study groups. Statistical significance was set at a *P* value of .05.

## Results

### Medicaid vs. private overall

Contact was made with 465 of 510 (91.2%) PT practices. Overall, 52.7% of the PT practices accepted Medicaid insurance, while 94.9% accepted private insurance (*P* < .0001, Table I). Of those practices not accepting insurance, the most commonly cited reason was that the practice did not have a contract with the insurance carrier (40.7%, Table II).

**Table I:** Comparison of insurance types accepted nationally.

	Private insurance (n = 466)		Medicaid insurance (n = 465)	
	Accepted	Not accepted	Accepted	Not accepted
Insurance overall	442 (94.9%)	24 (5.1%)	245 (52.7%)	220 (47.3%)
Average Yelp rating	4.72 ± 1.39	4.92 ± 0.82	4.66 ± 1.40	4.80 ± 1.30
Average median income for ZIP code of PT practice∗	58,802 ± 18,447 (15,363-140,500)	65,082 ± 24,957 (31,920-139,306)	57,099±17,057 (15,363-125,096)	61,481 ± 20,494 (16,662-140,500)
Median # days to 1st appointment∗	2, IQR: 1-4, (0-43)	N/A	2, IQR: 1-4, (0-72)	N/A

∗ Parentheses indicate range of values.

**Table II:** Ability to refer to another practice for care and reason for not accepting insurance among those practices who do not accept insurance plans.

	Private insurance (n = 24) (%)	Medicaid insurance (n = 465) (%)
Ability to refer to another practice?		
Yes	11 (45.8)	118 (53.9)
No	13 (54.2)	89 (40.6)
Reason for not accepting		
No contract	11 (45.)	88 (40.0)
Low payment	0 (0)	24 (10.9)
Cash pay only	12 (50)	10 (4.5)
Pediatric only	0 (0)	22 (10.0)
Secondary Ins. only	0 (0)	17 (7.8)
Unknown	1 (4.2)	59 (26.8)

The average Yelp rating was similar for practices accepting Medicaid insurance as compared with those accepting private insurance. However, the average household median income in the ZIP code of PT practices accepting Medicaid was significantly lower than for PT practices not accepting Medicaid insurance ($57,099 vs. 61,481, *P* = .01).

### Medicaid expansion states vs. nonexpansion states

Medicaid insurance was more likely to be accepted in a Medicaid expansion state as compared with a nonexpansion state (56.1% vs. 46.3%, *P* = .05, Table III). Private insurance was also more likely to be accepted in a Medicaid expansion state than a nonexpansion state (96.7% vs. 91.3%, *P* = .01). No contract was the most common reason for insurance not being accepted within both non-Medicaid expansion states (35%) and Medicaid expansion (44.8%) states. If the insurance type was not accepted, ability to refer to another PT practice accepting the insurance was also similar between non-Medicaid Expansion states (53.0%) and Medicaid expansion states (53.1%). Yelp rating and average median income based on ZIP code of PT location were also similar.

**Table III:** Comparison of insurance accepted among Medicaid expansion states vs. non-Medicaid expansion states.

	Medicaid expansion states		Nonexpansion states	
	Private (n = 306)	Medicaid (n = 305)	Private (n = 160)	Medicaid (n = 160)
% Accept Insurance∗	296 (96.7%)	171 (56.1%)	146 (91.3%)	74 (46.3%)
If no, referral to other practice	4 (40%)	72 (53.7%)	7 (50%)	46 (53.5%)
Reason for not accepting				
No contract	3 (30%)	61 (45.5%)	8 (57.1%)	27 (31.4%)
Low/No payment	0 (0%)	14 (10.4%)	0 (0%)	10 (11.6%)
Unknown	1 (10%)	35 (26.1%)	0 (0%)	24 (27.9%)
Pediatric	0 (0%)	8 (6.0%)	0 (0%)	14 (16.3%)
Secondary only	0 (0%)	11 (8.2%)	0 (0%)	6 (7.0%)
Cash pay only	6 (60%)	5 (3.7%)	6 (42.9%)	5 (5.8%)
Average time to first appt (days)	3.04 ± 3.29 (Range: 0-43, Median: 2, IQR: 1-4)	4.11 ± 7.14 (Range: 0-72, Median: 2, IQR: 2-4)	2.58 ± 2.14 (Range: 0-11, Median: 2, IQR: 1-3)	2.51 ± 1.81 (Range: 0-10, Median: 2, IQR: 1-3)
Average overall Yelp Rating	4.67 ± 0.62		4.63 ± 0.56	
Average median income of PT location ZIP code	$60,129 ± 19,618 (Range: $15,363-140,500)		$57,501 ± 17,258 (Range: $20,110-125,096)	

∗ *P* < .0001.

The time to first available appointment varied more in Medicaid expansion states (range: 0-43 days for private insurance; range: 0-72 days for Medicaid insurance) than in nonexpansion states (range: 0-11 days for private insurance; range: 0-10 days for Medicaid insurance). Despite a varied range of time to first appointment, the median number of days to first appointment was the same across both insurance types in Medicaid expansion states as compared with non-Medicaid expansion states (median: 2 days).

### Regional variations in PT practices

When evaluating PT practices by region, the West demonstrated the highest rates of Medicaid insurance acceptance (73.3%), while the South was found to have the lowest rates of Medicaid acceptance (32.3%, Table IV). Interestingly, the South also has the lowest rates of Medicaid expansion (41% of states) as compared with the Midwest (58%), West (77%), and Northeast (100%), which may further explain the low Medicaid acceptance rates in the South. Similarly, the South also demonstrated the lowest rates of private insurance acceptance among PT practices (90.6%). Of the practices not accepting insurance, the primary reasons varied by region. For example, 14.8% of practices in the South stated they only accepted Medicaid for pediatric patients (up to age 21 years), whereas this reason was not given for any practices in the Midwest or West regions. Cash payment only was also most common in the South (11.5%) and less common in the West (5.7%).

**Table IV:** Insurance acceptance by region in the United States.

	Midwest (n = 220) (%)	Northeast (n = 159) (%)	South (n = 317) (%)	West (n = 235) (%)
% Accept Medicaid	70 (63.6)	37 (46.8)	51 (32.3)	87 (73.7)
% Accept Private Ins	107 (97.3)	78 (97.5)	144 (90.6)	113 (96.6)
If no, referral to another practice	23 (59.0)	25 (61.0)	67 (58.3)	14 (41.2)
Reason not accepting				
No contract	15 (34.9)	22 (50.0)	48 (39.3)	14 (40)
Low payment/no payment	8 (18.6)	2 (4.5)	8 (6.6)	6 (17.1)
Unknown	14 (32.6)	11 (25.0)	26 (21.3)	9 (25.7)
Pediatric	0 (0)	4 (9.1)	18 (14.8)	0 (0)
Secondary only	3 (7.0)	2 (4.5)	8 (6.6)	4 (11.4)
Cash pay only	3 (7.0)	3 (6.8)	14 (11.5)	2 (5.7)

## Discussion

In evaluation of 465 PT practices across the United States, we found that significantly fewer practices accept Medicaid insurance as compared with private insurance (52.7% vs. 94.9%, respectively, *P* < .0001). This suggests that patients with Medicaid insurance may have greater difficulty accessing PT services after RCR in the United States as compared with patients with private insurance. While Medicaid insurance was more likely to be accepted in a Medicaid expansion state as compared with a nonexpansion state (56.1% vs. 46.3%, *P* = .05), this finding was only borderline statistically significant, thus indicating that patients in Medicaid expansion states are still having difficulties gaining access to PT services after RCR despite significant PPACA efforts to expand government insurance coverage to improve access to care.

Consistent with our findings, prior studies in the orthopedic literature have concluded that Medicaid insurance is less likely to be accepted compared with private insurance. Patterson et al^6^ found that the likelihood of being able to make an appointment for PT to manage an acute rotator cuff tear in North Carolina was 8.8 times higher (95% CI: 2.5%-31.5%) for patients who were privately insured compared with patients with Medicaid insurance. Similarly, Rogers et al^7^ found that in the Greater Boston Area, only 51.4% of practices accepted Medicaid insurance, while 96.4% accepted private insurance (*P* = .02) for PT appointments after RCR. This is similar to the national acceptance rates we have found in this study with only 52.7% of practices accepting Medicaid vs. 94.9% accepting private insurance. The authors further reported that, among PT practices accepting Medicaid insurance, the average time to first available appointment was significantly longer for Medicaid patients vs. private payors (8.3 vs. 6.3 days, *P* = .001). In contrast, we found the median time to first appointment to be similar between patients with Medicaid (2, IQR: 1-4) and private insurance (2, IQR: 1-4); however, the range of time to first appointment was greater for patients with Medicaid insurance (0-72 days) vs. private insurance (0-43 days). Furthermore, based on US census data for the ZIP code in which each PT practice was located, we found the average household median income was significantly lower for PT practice ZIP codes accepting Medicaid ($57,099) as compared to those accepting private insurance ($61,481) with a *P* value of .01. Therefore, it appears that Medicaid-accepting PT practices tend to be located in lower-income neighborhoods.

Although many PT practices stated having “no contract” was the primary reason for not accepting Medicaid insurance, the response provides little insight into why such a contract has yet to be established. Labrum et al^2^ reported that patients with Medicaid insurance in expansion states continue to experience greater difficulty obtaining access to orthopedic care as compared with patients with commercial insurance. However, they found that access to care for patients with Medicaid insurance significantly increased in correlation with higher reimbursement rates (*P* < .001). Therefore, increased payment by Medicaid insurance plans must be considered to increase incentives for PT practices to establish contracts with Medicaid insurers. In addition, in North Carolina, Medicaid beneficiaries are only entitled to 1 therapy visit per year between physical, occupational, and speech therapy. The exception to this rule is in the postoperative period, which will allow a patient access to 1 PT evaluation and 3 treatment visits after RCR. However, more visits are allowed (2 PT evaluations and 8 treatments) within a specific time frame for those patients undergoing joint replacement surgery. These postoperative visit restrictions are often not sufficient to allow optimal recovery with additional visits requests reviewed on a case-by-case basis. These Medicaid limitations have led many PT facilities to refuse Medicaid contracts, thus effectively closing the door to appropriate postoperative PT access for Medicaid beneficiaries. Furthermore, most patients will need 3 to 6 months of formal PT after RCR to regain appropriate range of motion to optimize outcomes. Therefore, patients with Medicaid insurance are at an increased risk for poor postoperative outcomes owing to the amount of restrictions placed on the number of postoperative PT visits afforded them.

The results of this national study also identified a number of methods to restrict Medicaid access to PT services in certain states, which is likely tied to decreased reimbursement rates and greater patient complexity in the Medicaid population. For example, in Virginia and Oklahoma, PT practices stated that Medicaid was only accepted at hospitals and not in the outpatient clinic settings. For patients who live a significant distance from a hospital or lack adequate transportation, these types of regulations may prove prohibitive for patients with regards to access to care. For many practices contacted in Maine and Washington State, the practice stated that Medicaid insurance is accepted but is limited to only two patients per practice at a given time. Although speculation, the reasons behind these restrictions are likely owing to low reimbursement rates.

In terms of Medicaid expansion, the PPACA was developed in 2010 with the goal of increasing access to health care to millions of Americans. Although more individuals have been able to obtain access to Medicaid insurance, 1 decade later, we found that access to postoperative care is still limited with only 56.1% of Medicaid patients having access to PT services after RCR in Medicaid expansion states and 46.3% in nonexpansion states. This calls into question whether health insurance coverage necessarily equates to access to health care. Nguyen et al^4^ found that despite the expansion of Medicaid coverage, only 38% of orthopedic surgeons will schedule an appointment for a Medicaid insured 11-year-old child with a distal radius fracture compared with 83% for a privately insured child with the same fracture. Interestingly, patients with Medicaid insurance in states without Medicaid expansion were more successful in obtaining appointments than Medicaid patients in expansion states (47% vs. 30%). In addition, we found that the range of time to first appointment was longer for both private and Medicaid insured patients in Medicaid expansion states as compared with nonexpansion states. Encouragingly, despite the longer range of wait times in Medicaid expansion states, this was not related to satisfaction of care received as the overall Yelp ratings were similar between expansion and nonexpansion states. The longer range of time to first appointment among Medicaid expansion states may be due to the fact that the PT workforce has not expanded to sufficiently accommodate the increased patient demand because of the PPACA. This finding would suggest the need to train additional healthcare workers to meet the increased demand.

The findings of this study have important implications for orthopedic surgeons, particularly those who see a substantial proportion of Medicaid patients with rotator cuff tears or work in safety net hospitals. Access to care based on insurance type should be considered for all patients undergoing RCR, and orthopedic surgeons should counsel all patients on the importance of early identification of PT services to avoid delays in rehabilitation. By properly identifying patients with difficulty accessing PT services owing to their insurance status, alternative rehabilitation options can be considered including the use of self-directed home exercises, enlisting family members to help with the postoperative rehab, or possibly seeking out PT practices in an area interested in doing pro bono work. Given the significant number of PT facilities not accepting Medicaid patients after RCR, there is a significant concern that patients with Medicaid insurance will be disadvantaged in their postoperative recovery as compared with patients with private insurance. Finally, while other smaller commercial insurance providers and Medicare were not directly studied here, orthopedic surgeons should be mindful that these patient populations may also experience similar limitations.

The strengths of this study are in the evaluation of access to PT services on a national level with further comparison of Medicaid expansion vs. nonexpansion states. We also were able to evaluate the relationship between insurance type accepted, the average household median income for a particular PT practice via ZIP codes and Yelp rating. Moreover, our ability to use identical mock patients allows for significant reproducibility that would not be available with actual patients. Limitations include the inability to clearly decipher reasons for not accepting Medicaid insurance as most practices simply stated there was “no contract” with Medicaid or the reason for not accepting was “unknown.” The reason for not having a contract for Medicaid may be helpful to make further recommendations to resolve the discrepancies in access to care between those with private vs. Medicaid insurance. In addition, we only evaluated PT services in state capitals, which may not provide insight into differences in access to care between urban vs. rural practices as there is some evidence to suggest that patients with Medicaid insurance in urban areas have greater difficulty obtaining PT appointments as compared with those in rural settings.^5^ Furthermore, the use of the APTA Website to identify PT practices in state capitals may have had an impact on the physical therapy practices identified because practices are only listed on the Website if a physical therapist is a member of the APTA at a given practice. The use of mock patients in and of itself is also a limitation considering these patients are unable to actually be scheduled or have continuing dialog as to the status of their insurance acceptance.

Access to PT services after RCR is significantly lower for patients with Medicaid insurance as compared with those with private insurance. Access to PT services in Medicaid expansion states remains low and is not substantially improved as compared to Medicaid nonexpansion states. As such, orthopedic surgeons should counsel their patients with Medicaid insurance to seek out PT services as early as possible in the postoperative period to avoid delays in rehabilitation. Alternative rehabilitation options should also be considered in those practices treating patients with Medicaid insurance to optimize postoperative outcomes given the lack of access to formal PT services.

## Conclusion

Overall, we found that significantly fewer PT practices accepted Medicaid insurance nationally compared with private insurance, which suggests that those patients with Medicaid insurance have greater difficulty accessing physical therapy services after rotator cuff repair in the United States compared with patients with private insurance. While Medicaid insurance was more likely to be accepted in a Medicaid expansion state as compared with a nonexpansion state, this finding was only borderline significant, which indicates that patients in Medicaid expansion states are still having difficulty accessing physical therapy services despite ACA efforts to expand insurance coverage to help improve access to care to a larger population. Orthopedic surgeons should counsel their patients with Medicaid insurance to seek out physical therapy services as early as possible in the postoperative period to avoid delays in rehabilitation. Alternative rehabilitation options should also be considered in practices treating patient populations predominantly with Medicaid insurance, including the use of home therapy or family-assisted therapy protocols.

## Disclaimers:

*Funding:* No funding was disclosed by the authors.

*Conflicts of Interest:* The authors, their immediate families, and any research foundations with which they are affiliated have not received any financial payments or other benefits from any commercial entity related to the subject of this article.
