# Supplementary material for: National disparities in access to physical therapy after rotator cuff repair between patients with Medicaid vs. private health insurance
Source: JSES Int. 2021 Jan 16;5(3):507–11. doi: 10.1016/j.jseint.2020.11.006 (PMC8178595; doi:10.1016/j.jseint.2020.11.006)
Supplement: Appendix I [file mmc1.docx]

**Appendix 1**

**Telephone Script:**

**Study Coordinator (SC)**: Hello, my name is XXX. I just had an arthroscopic rotator cuff repair surgery one week ago and I saw my orthopaedic surgeon today for my first postoperative visit. My surgeon would like me to start my physical therapy as soon as possible and I have a PT script with his/her protocol. I have Medicaid **or** Blue Cross Blue Shield PPO; do you take my insurance?

**Response:** Yes or No (RECORD)

*If yes…*

**SC:** Great, thank you for this information.  When is your next available appointment?

RECORD: 1. Day you called, 2. Day of next available appointment

**SC:** I’ll have to check my schedule, and I will call back to let you know what date and time works for me to schedule an evaluation.

*If no…*

**SC:**  Okay, why do you not accept my insurance?

RECORD: Reason (verbatim)

**SC:**Can you provide me with the contact information of another PT clinic that will take my insurance?

RECORD: Yes or No
